# Association of Pulmonary Valve Morphology Differences With Outcomes in Tetralogy of Fallot Repair With Right Ventricular Outflow Tract Incision

**DOI:** 10.3389/fcvm.2021.695876

**Published:** 2021-08-04

**Authors:** Jinyang Liu, Xianchao Jiang, Bo Peng, Shoujun Li, Jun Yan, Qiang Wang, Zhimin Liu

**Affiliations:** ^1^Center for Pediatric Cardiac Surgery, Fuwai Hospital, National Center for Cardiovascular Diseases, Chinese Academy of Medical Sciences and Peking Union Medical College, Beijing, China; ^2^Department of Cardiac Surgery, Yunnan Fuwai Cardiovascular Hospital, Beijing, China; ^3^Department of Cardiology, Fuwai Hospital, National Center for Cardiovascular Diseases, Chinese Academy of Medical Sciences and Peking Union Medical College, Beijing, China

**Keywords:** tetralogy of fallot, tricuspid pulmonary valve, bicuspid pulmonary valve, unicuspid pulmonary valve, right ventricular outflow tract incision

## Abstract

**Background:** Current observational studies may not have large samples to investigate the relationship between pulmonary valve (PV) morphology differences and outcomes after complete repair for tetralogy of Fallot (TOF) by right ventricular outflow tract (RVOT) incision. This study aimed to assess the impact of PV morphology differences on outcomes after complete repair for TOF.

**Methods:** This is a retrospective cohort study. Consecutive patients who underwent TOF repair with RVOT incision at Fuwai Hospital from January 2012 to December 2017 were included and compared according to PV morphology differences (unicuspid or bicuspid was abnormal morphology, while the tricuspid valve was normal morphology). The primary outcome was defined as a composite of death, or reintervention, or significant annular peak gradient (APG), or significant pulmonary regurgitation (PR), whichever occurred first. Multivariable Cox model analysis was used to assess the relationships between PV morphology differences and outcomes. Subgroup analysis and Propensity-score analysis were performed as sensitivity analyses to assess the robustness of our results.

**Results:** The cohort included a total of 1,861 patients with primary diagnosis of TOF, with 1,688 undergoing CR-TOF with RVOT incision. The median age was 318 days [interquartile range (IQR): 223–534 days], a median weight of 8.9 kg (IQR: 7.6–10.5 kg) and 60.0% (1,011) were male. Complete follow-up data were available for 1,673 CR-TOF patients with a median follow-up duration of 49 months. Adjusted risks for the primary outcome and significant APG were lower for patients with normal PV morphology at follow up [adjusted hazard ratio (HR): 0.68; 95% CI: 0.46–0.98; adjusted HR: 0.22; 95% CI: 0.07–0.71, respectively]. The trend for the primary outcome during follow-up remained unchanged, even in subgroups and propensity score matching analyses.

**Conclusions:** In this analysis of data from a large TOF cohort, patients with normal tricuspid PVs were associated with a decreased risk of the primary outcome and a lower risk of significant APG, as compared with patients with abnormal unicuspid or bicuspid PVs.

## Introduction

Tetralogy of Fallot (TOF) is the most common cyanotic congenital heart disease (1, 2) and involves ventricular septal defect, pulmonary valve (PV) stenosis, an overriding aorta, and right ventricular hypertrophy. Recent years have seen the progression of TOF repair. The debates about type and timing of repair (3–8), the concerns about preservation of PV (9), pulmonary valve annular (PVA) (10–14), and infundibulum (15), and so forth, are still heatedly discussed among people. However, abnormal PV morphology is a common clinical problem in TOF population, and more than 80% of patients make up this abnormal population (including unicuspid and bicuspid valve) (16, 17). In the initial complete repair for TOF, intraoperative PV plasty seems inevitable. The fibrotic thickening at the leaflet free edge, abnormal histologic PV specimens and PV dysplasia were significantly higher in patients with abnormal PV morphology (unicuspid and bicuspid valve) compared to those with normal tricuspid valve morphology (16). This abnormal PV morphology raises an intriguing question: is long-term PV function after completely repaired TOF driven by a morphology factor rather than surgical strategies? Yet, the trend for the impact of PV morphology differences on PV function was rarely reported from large TOF series.

To directly assess the impact of PV morphology differences, we performed this study deriving from a large TOF sample in China, which would provide a novel insight to the assessment of prognosis of repaired TOF. We referred to Jeon and his colleagues' brand-new definition for the end point of TOF repair to comprehensively evaluate the PV function (8).

## Materials and Methods

This is a retrospective cohort study. The Medical Ethics Review Committee of Fuwai Hospital approved this study (No. 2020-1318). Informed consent was waived. This study was registered at www.chictr.org.cn (ChiCTR2000033234).

### Data Collection

**Figure 1:**
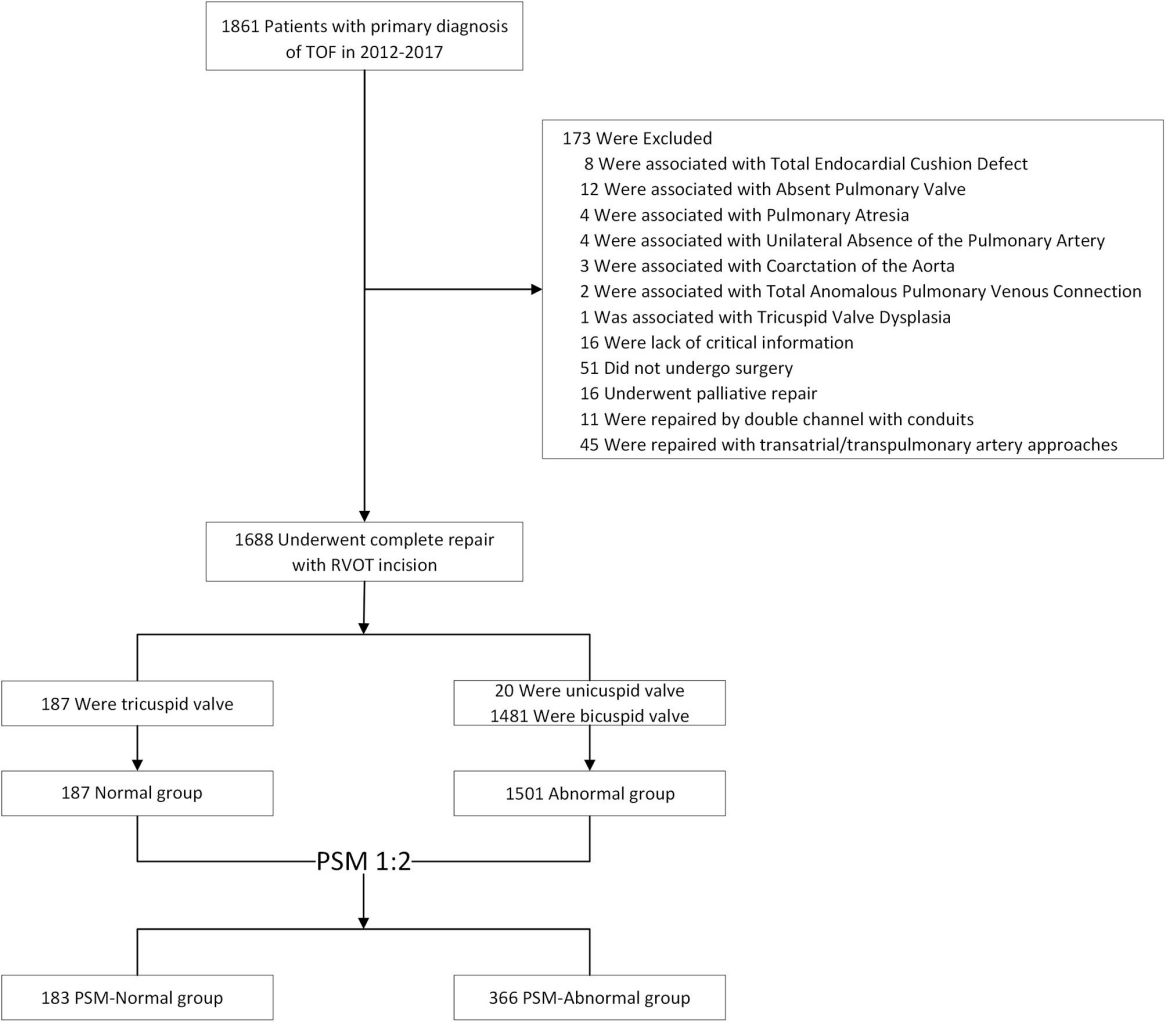
Cohort assembly. Final cohort derivation for pulmonary valve morphology differences after the application of study eligibility criteria.

The data obtained included baseline demographic details, preoperative and intraoperative information, and surgical techniques involved. Follow-up was performed via office visits or telephone contact. The analysis excluded any patient with a primary diagnosis or secondary diagnosis that included any of the complicated cardiac malformations, or undergoing palliative repair or complete repair without right ventricular outflow tract (RVOT) incision. More inclusion and exclusion criteria were shown in Figure 1.

### Variables and Definitions

Echocardiogram results were obtained by using standard views recommended by the published guideline (18) and reviewed for degree of pulmonary regurgitation (PR) and annular peak gradient (APG). Pulmonary valve annulus (PVA) z-score was calculated according to the previous study (18). Pulmonary valve morphology was evaluated according to preoperative echocardiogram results and intraoperative reviews, including unicuspid, bicuspid, and tricuspid. The tricuspid morphology was normal while the unicuspid or bicuspid valve was abnormal PVs. One-stage repair was defined as a patient who had complete repair for TOF as initial operation. Staged repair was defined as a patient who had complete repair for TOF following prior palliative repair. Patients with uncut PVAs were regarded as annulus-sparing (AS). Patients undergoing a return to bypass during index operation were defined as repump. We defined an experienced operator as an operator who performed at least 20 cases of complete repair for TOF per year for at least 3 consecutive years between 2012 and 2017. Pulmonary valve specific-technical performance score (PV-TPS) (19) was introduced as a standard to evaluate surgical adequacy. Pulmonary valve specific-technical performance score as class 1 indicated APG <20 mmHg or none/trivial PR, class 2 was APG between 20 and 40 mmHg or mild/mild to moderate PR, and class 3 indicated APG > 40 mmHg or moderate or greater PR (19).

### Objectives and Study End Points

The study aimed to investigate the impact of PV morphology on clinical outcomes after complete repair for TOF. The primary end point was a composite of catheter or surgical reintervention, or significant ROVT obstruction (APG-TPS in class 3), or significant PR (PR-TPS in class 3), whichever occurred first at follow up. Secondary outcomes were the individual events of the primary end point.

### Surgical Techniques

On cardiopulmonary bypass (CPB) via ascending aortic and bicaval cannulation, and under cardioplegic arrest and moderate hypothermia, the infundibulum was incised longitudinally to just below the level of the PVA in all patients. Parietal muscle bundle was resected and ventricular septal defect was closed. Patched enlargement with fresh autologous pericardium would be adopted in main pulmonary artery (MPA) and branch pulmonary artery when necessary. Valvuloplasty is commonly a combination of splitting of fused commissures extending to the level of PVA to achieve larger effective orifice. In patients with severely dysplasia PVs, we used continued T-shaped fashion and inverted T-shaped fashion in infundibulum and MPA incision, extending parallel to the annulus, respectively, to release sub- and supra-valvular tissues which might restrict the PVA growth as possible as we can (Supplementary Figures 1A,C). In some cases with bicuspid PVs, longitudinal incision along the middle line of one or two leaflets to the level of PVA would be performed to release constraints on PVA if the effective orifice was still too small after above-mentioned operations, and PV-plasty with a large triangular fresh autologous pericardium patch would be conducted to prevent PVR and orifice restenosis by stretching circumference of PVA to achieve orifice area increasing (Supplementary Figure 1B). After above-mentioned techniques adopted, if the PVA is still not fully extended, the transannular patch will be required.

### Statistical Analysis

Numerical data are presented as mean ± standard deviation (SD) or median with range [interquartile range (IQR)] and were analyzed by the Mann-Whitney U test or Kruskal-Wallis test. Qualitative data are presented as percentages and were compared using the chi-square test or Fisher's exact test, where appropriate.

Cox proportional-hazards regression models were used to investigate the association between PV morphology differences and end-point events. Baseline variables that had a *P* < 0.1 in univariable models were introduced as covariates in a multivariable model with a stepwise forward method (nine covariates, namely, age, height, preoperative saturation, PVA diameter, PVA z-score, RVOT gradient, operator experience, surgical strategies, and staged operation) (Supplementary Table 1). The adjusted hazard ratios (HRs) for PV morphology were estimated in reference to the abnormal group. Hazard ratios are presented with 95% confidence intervals (CIs). The proportional-hazards assumption was tested by inspection of Schoenfeld Residuals. We also explored the relationship between PV morphology and the composite primary outcome in subgroups. In addition, given differences in baseline characteristics between two group (Table 1), we conducted propensity-score methods to reduce the effects of confounding. The individual propensities for patients with normal PV were estimated with the use of a multivariable logistic-regression model that included sex, age, weight, PVA diameter, preoperative saturation, surgical strategies, and staged operation. Propensity-score matching (PSM) was performed with a 1:2 matching protocol without replacement (greedy-matching algorithm), with a caliper width equal to 0.01 on the propensity score scale. Standardized differences were estimated for all the baseline covariates before and after matching to assess prematch imbalance and postmatch balance (Supplementary Table 2). Associations between PV morphology differences and the primary outcome were then estimated by multivariable Cox regression model (Supplementary Table 4).

**Table 1:** Baseline characteristics of patients with TOF.

**Patients characteristic**	**Normal PV**	**Abnormal PV**	***P*-value**
	**(*N* = 187)**	**(*N* = 1,501)**	
Male sex, no. (%)	110 (58.8)	901 (60.6)	0.75
Age, days	311 (220–674)	318 (224–524)	0.39
Height, cm	78.4 ± 18.2	75.2 ± 14.3	0.006
Weight, kg	8.7(7.8–10.8)	9.0 (7.6–10.5)	0.80
Preop. SaO_2_, %	85.9 ± 7.7	83.4 ± 9.1	<0.001
PVAD, mm	9.9 ± 3.0	8.6 ± 2.4	<0.001
PVA Z-score	−1.7 (−2.4–0.9)	−2.3 (−2.8–1.6)	<0.001
Pre-op. RVOT peak gradient, mmHg	71.1 ± 18.9	74.4 ± 16.5	0.011
Procedures for complete repair			<0.001
AS repair, no. (%)	158 (84.5)	787 (52.4)	
TAP repair, no. (%)	29 (15.5)	714 (47.6)	
Staged repair, no. (%)	5 (2.7)	56 (3.7)	0.47
Additional procedures			
MPA plasty, no. (%)	55 (29.4)	872 (58.1)	<0.001
LPA plasty, no. (%)	11 (5.9)	212 (14.1)	0.002
RPA plasty, no. (%)	4 (2.1)	48 (3.2)	0.43
PV plasty, no. (%)	0 (0.0)	24 (1.6)	0.08
MAPCAs closure, no. (%)	4 (2.1)	92 (6.1)	0.026
^*^Operator experience			0.84
Experienced operator, no. (%)	132 (70.6)	1,070 (71.3)	
Less experienced operator,	55 (29.4)	431 (28.7)	
no. (%)			
Follow-up duration, m	49 (31–63)	49 (36–64)	0.13

*Data are presented as n (%), mean ± standard deviation, or median [interquartile range (IQR)]. AS, annular-sparing; TAP, transannular patch; Pre-op, preoperative; PVA, pulmonary valve annulus; PVAD, pulmonary valve annulus diameter; ASD, atrial septal defect; PDA, patent ductus arteriosus; RVOT, right ventricular outflow tract; MPA, main pulmonary artery; LPA, left pulmonary artery; RPA, right pulmonary artery; MAPACs, major aortopulmonary collateral arteries*.

* *The operator experience is defined according to volume of complete repair of TOF they performed each year between 2012 and 2017*.

The primary analysis used multivariable Cox regression models. We conducted secondary analyses that used PSM with multiple Cox regression and subgroup analyses.

The statistical analyses were performed with the use of the statistical software packages R (http://www.R-project.org, The R Foundation) and EmpowerStats (http://www.empowerstats.com, X&Y Solution, Inc., Boston, MA) and SPSS version 26.

## Results

From January 2012 to December 2017, a total of 1,688 patients fulfilled the inclusion criteria and underwent TOF repair by RVOT incision. Of these, 1,501 (88.9%) had abnormal PVs, and 187 had normal ones. The median age was 318 days (IQR: 223–524 days), a median weight of 8.9 kg (IQR: 7.6–10.5 kg), and 60.5% were male. No death occurred during the follow-up. Of 1,688 patients, 1,638 (97.9%) have completed at least 1-year follow-up and 1,174 (70.2%) have been followed up for 3 year.

### Patient Characteristics

The characteristics of the patients categorized by PV morphology differences are listed in Table 1, both in the unmatched and propensity-score-matched analytic samples. In the unmatched sample, TOF patients with normal PVs were higher than the abnormal ones, and were more likely to have higher preoperative saturation, lower preoperative RVOT gradients, larger PVA diameter, and PVA z-score after adjustment for weight and height. The higher rate of AS repair was found in the normal group, while higher rates of TAP repair, MAP and left pulmonary artery plasty were found in the abnormal group. In the matched analytic sample, 183 patients had normal PVs and 366 did not have. The differences of baseline variables between PV morphology groups were attenuated in the propensity-score-matched samples as compared with the unmatched (Supplementary Table 2).

### Clinical Outcomes Between Two Groups Before and After Matching

Among the 1,688 patients included in the analysis, in-hospital deaths occurred in 15 patients (2 patients in the normal group, and 13 in the abnormal group). In the unmatched sample, TOF patients with normal PVs were more likely have shorter ventilation duration (*p* < 0.001), ICU stay (*p* = 0.012), and a lower risk for significant PR (moderate or greater PR) (*p* = 0.018) at discharge. Among the patients with abnormal PVs, 27 (1.8%) required reintervention at follow up. And there were significant differences in adequacy of PV repair, both in TPS for APG and PR, at follow up. In addition, patients with abnormal PVs were more likely to have a higher risk of a primary outcome. In the matched sample, the differences of clinical outcomes between two groups were attenuated in the propensity-score-matched samples as compared with the unmatched. However, there were significant differences in adequacy of PV repair, and patients with abnormal PVs had a higher trend in the incidence of a primary end point. More details were shown in Table 2 and Supplementary Table 2.

**Table 2:** Clinical outcomes of patients with CR-TOF.

**Patients characteristic**	**Normal PV**	**Abnormal PV**	***P*-value**
	**(*N* = 187)**	**(*N* = 1,501)**	
Crossclamp time, min	73.4 ± 26.2	75.9 ± 26.7	0.24
^&^Repump, no. (%)	1 (0.5)	25 (1.7)	0.24
Ventilation duration, h	13 (8–23)	18 (10–28)	<0.001
ICU stay duration, days	2 (1–4)	3 (1–4)	0.012
Postop length of stays, days	8 (7–12)	9 (7–12)	0.15
In-hospital death, no. (%)	2 (1.1)	13 (0.9)	0.78
APG in TPS Class 3 at discharge, no. (%)	8 (4.3)	96 (6.4)	0.25
PR in TPS Class 3 at discharge, no. (%)	11 (5.9)	174 (11.6)	0.018
Reintervention at follow up, no. (%)	0 (0.0)	27 (1.8)	0.07
^*^Adequacy of PV repair at follow up			
TPS for APG			0.002
Class 1 (APG <20	116 (62.7)	841 (56.5)	
mmHg), no. (%)			
Class 2 (APG 20–40	66 (35.7)	512 (34.4)	
mmHg), no. (%)			
Class 3 (APG > 40	3 (1.6)	135 (9.1)	
mmHg), no. (%)			
TPS for PR			<0.001
Class 1 (none/trivial PR),	89 (48.1)	449 (30.2)	
no. (%)			
Class 2 (mild/mild-moderate	68 (36.8)	644 (43.3)	
PR), no. (%)			
Class 3 (moderate or	28 (15.1)	395 (26.6)	
greater PR), no. (%)			
TR Moderate or greater at follow up, no. (%)	2 (1.08)	17 (1.14)	0.94
^#^Primary outcome at follow up	29 (15.7)	486 (32.7)	<0.001

*Data are presented as n (%), mean ± standard deviation, or median [interquartile range (IQR)]. ECMO, corporeal membrane oxygenation; ICU, intensive care unit; postop, postoperative; TPS, technical performance score; APG, peak annular gradient; PR, pulmonary regurgitation; Other abbreviations as above*.

& *Repump means patients required a return to bypass during index operation*.

* *Patient numbers in adequacy of PV repair at follow up excluded in-hospital deaths*.

# *The primary outcome was a composite of reintervention or TPS in Class 3 (APG > 40 mmHg or moderate or greater PR)*.

### Study End Points

Over a median follow-up of 49 months, 515 (30.8%) had a primary end-point event (27 patients underwent reintervention, 138 had APG in TPS 3, and 423 had PR in TPS 3). In the crude, unadjusted analysis, patients with normal PVs were less likely to have a risk of a primary end-point event or significant APG than those with abnormal PVs (HR: 0.45; 95% CI: 0.31–0.66; HR: 0.17; 95% CI: 0.05–0.53, respectively) (Table 3). In the multivariable Cox analyses, there was significant association between PV morphology differences and the composite primary outcome or significant APG (adjusted HR: 0.68; 95% CI: 0.46–0.98; adjusted HR: 0.22; 95% CI: 0.07–0.71, respectively) (Table 3).

**Table 3:** Association between pulmonary valve morphology and follow-up outcomes in the crude analysis and multivariable analysis.

**Analysis**	Primary outcome^*^	**Significant APG**
No. of events/no. of patients at risk (%)		
Normal PV	29/185 (15.7)	3/185 (1.6)
Abnormal PV	486/1,488 (32.7)	135/1,488 (9.1)
Crude analysis—hazard ratio (95% CI)	0.45 (0.31, 0.66)	0.17 (0.05, 0.53)
Multivariable analysis—hazard ratio (95% CI)	0.68 (0.46, 0.98)	0.22 (0.07, 0.71)

*Events (%) and HRs from adjusted analyses are presented. HRs were calculated in reference to the abnormal PV group*.

*Cox model adjusted for: age, height, preoperative saturation, PVA diameter, PVA z-score, RVOT gradient, operator experience, surgical strategies, and staged operation*.

*CI, confidence interval; HR, hazard ratio; Other abbreviations as above*.

* *The primary outcome was a composite of reintervention or TPS = Class 3 (APG > 40 mmHg, moderate or greater PR, or both)*.

**Figure 2:**
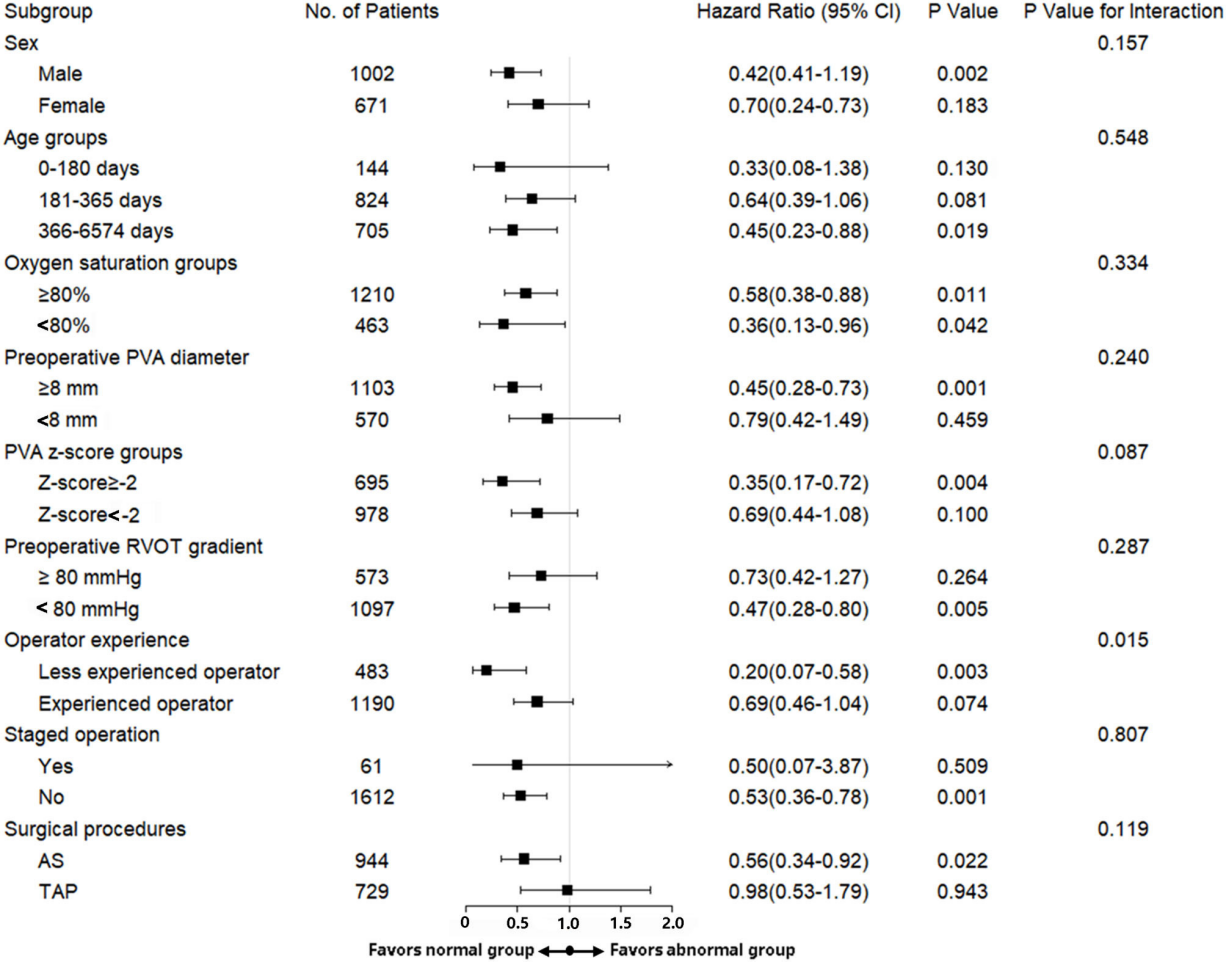
Multivariable Cox regression results between normal pulmonary valve morphology and abnormal pulmonary valve morphology for the composite primary end point among patient subgroups. Multivariable Cox regression results for a composite end point of reintervention and PV-TPS in Class 3 at follow up among patient subgroups including different sex groups, age groups, PVA z-score groups, PVAD groups, and whether AS was used and whether staged operation was used.

In subgroup analyses, the associations of PV morphology differences with the primary end point in TOF patient with sex groups, different age groups, preoperative saturation, PVA diameter, PVA z-score groups, preoperative RVOT gradient, operator experience, whether staged operation was used and whether AS was used were also consistent with the overall results. We found no significant difference in the magnitude of effect of PV morphology on the risk of the primary end point at follow up according to sex (*p* = 0.16), age (*p* = 0.55), preoperative saturation (*p* = 0.33), PVA diameter (*p* = 0.24), PVA z-score (*p* = 0.09), preoperative RVOT gradient (*p* = 0.29), whether staged operation was used (*p* = 0.80), and whether AS was used (*p* = 0.12) (Figure 2). An additional PSM analysis yielded similar results (Supplementary Table 4).

## Discussion

In this analysis involving a large sample of consecutive patients with TOF undergoing complete repairs, the risk of a composite primary end point and significant APG was significantly lower among patients with normal PV morphology. With the analytic approaches we used in this examination of our observational cohort, we have tried to minimize possible confounding in a variety of ways. Irrespective of PVA diameter, PVA z-score, operator experience, and surgical technique, the results emphasized the importance of close follow-up in patients with abnormal PVs after complete repair for TOF. Furthermore, during the same follow-up, the abnormal cases developed more significant PR (TPS in Class 3) (26.6% in the unmatched sample and 18.3% in the matched sample) compares with that at discharge (11.6% in the unmatched sample and 7.7% in the matched sample), suggesting that PV leaflet growth was not matched with annular growth.

As we noted in the introduction, the findings from an early study including a small series of TOF patients showing mucoid dysplasia with fibrotic thickening at the leaflet free edge in 49% of PV specimens (16, 20). Besides, the authors found that cases with unicuspid and bicuspid valves were associated with a higher risk for dysplasia leaflets compared to those with normal valves (16, 20). Additionally, patients with dysplastic PV leaflets were associated with a risk for earlier onset PR (16, 20). Our results were consistent with it. In addition, their experience with valve-sparing repair demonstrated that abnormal valve morphology was associated with a higher risk of reintervention for re-stenosis (16, 20). We also found that a higher rate of midterm significant APG in patients with abnormal PVs, either in the unmatched cohort (9.1%) or the matched cohort (5.8%), compared with the normal cohort. As the authors pointed out, there is an important interaction between native PV leaflet biology and morphology that may have a strong effect on long-term PV function in TOF (20). In addition, we observed that although the higher rate of significant APG at discharge (4.3% in the unmatched cohort and 4.4% in the matched cohort) in the normal group had decreased over time (1.6% in the unmatched cohort and 1.7% in the matched cohort), the rate of significant PR at follow up (15.1% in the unmatched cohort and 15.5% in the matched cohort) had increased compared with that at discharge (5.9% in the unmatched cohort and 6.0% in the matched cohort). These findings suggest that surgical techniques may ensure a short-term satisfactory in PV function, but even in patients with normal PVs and early excellent PV behavior, eventual deterioration of PV function is common, and long-term PV performance is not changed by pure surgical interventions. Furthermore, the utilization rate of AS strategy in TOF repair is getting higher and higher, but it seems that cases with normal PVs would benefit more from the AS use. However, this study have shown that the vast majority of PVs in TOF were abnormal valves (especially bicuspid valve). In our clinical experience, the cases with unicuspid morphology are rare and this subgroup seems to benefits more from the tansannular patch (TAP) strategy. Therefore, we need to rethink the reasonableness of AS use in this abnormal subgroup. And it is important to comprehensively evaluate the PV anatomy and features, and a patient-tailored strategy may be required.

Additionally, in this study, the surgical age in our TOF cohort was slightly older (21, 22), with highest proportion of patients at 6 months to 1 year as high as 49.2% and at 1 year to 18 years as high as 42.1%. However, in subgroup analyses (Figure 2), the primary outcome remained unchanged across age categories. And considering the potential impact of different operators on the outcomes, we first introduce the operator experience, according to volume of complete repair of TOF they performed each year between 2012 and 2017, into multivariate model and subgroup analyses, in which the trend for the primary outcome remained similar.

## Strengths and Limitations

Our study has potentially important clinical implications. This study is based on a large TOF cohort and provides a novel insight into a risk factor of PV dysfunction. We show that PV morphology abnormality appears to be important to assess preoperatively, and patients with abnormal PVs require closer specialized medical care after repaired TOF. Furthermore, our results suggest that a higher risk of significant APG in population with abnormal PVs calls for an exquisite surgical procedure for abnormal PVs. In light of our findings, prospective validation of this concept is warranted and a patient-tailored management strategy should be developed.

Nevertheless, this study has some limitations. First, the retrospective nature of the study causes common biases such as information bias almost inevitable. Second, this was a single-center study. Its external validity is uncertain. Although we adjusted the analyses for known risk factors, we cannot rule out the presence of unmeasured confounders. However, our result arouses attention on PV morphology. Third, the follow-up time is short, and the emergence of robust endpoints (death or reintervention) often takes longer to be observed. As a result, it is hard to determine which group will benefit more. Fourth, we did not exclude severe TOF in this study, but the PV z-score is relatively high compared to the reported TOF spectrum, resulting from the z-score calculation. The reported formula we adopted in this study was based on the North American data (18), which is quite different from the Chinese population according to our experience. Therefore, we hope to standardize the calculation of z-score based on the characteristics of Chinese people in the future. Fifth, due to children rarely having the opportunity to undergo early screening and find the disease in our country, the children included in this study were slight older than encountered in other cohorts available in literatures. Given the low percentage of prenatal diagnosis and late presentation compared to other studies, some patients (even the most severe cases) might die before the diagnosis. As a result, we might exclude a group of severe TOF with the hypoplastic annulus.

Consequently, we have conducted a registered ambispective cohort study at www.chictr.org.cn (ChiCTR2000033234) to further address the limitations above.

## Conclusion

In our analysis involving a large sample of consecutive patients who underwent complete repairs by RVOT incision, those with normal PV morphology were associated with a significantly lower risk of the composite primary outcome and significant APG at follow up. Those with abnormal unicuspid or bicuspid PVs should be paid closer attention to after complete repairs. However, given the retrospective design, a perspective study should be conducted to further investigate this result.

## Data Availability Statement

The original contributions presented in the study are included in the article/Supplementary Materials, further inquiries can be directed to the corresponding author/s.

## Ethics Statement

The studies involving human participants were reviewed and approved by Medical Ethics Review Committee of Fuwai Hospital. Written informed consent to participate in this study was provided by the participants' legal guardian/next of kin.

## Author Contributions

JL was a major contributor in writing the original draft, data curation, and formal analysis. XJ and BP were responsible for data curation, formal analysis, and investigation. JY finished methodology. SL was responsible for funding and resources acquisition. QW and ZL was responsible for funding acquisition, supervision, conceptualization, and project administration. All authors read and approved the final manuscript.

## Conflict of Interest

The authors declare that the research was conducted in the absence of any commercial or financial relationships that could be construed as a potential conflict of interest.

## Publisher's Note

All claims expressed in this article are solely those of the authors and do not necessarily represent those of their affiliated organizations, or those of the publisher, the editors and the reviewers. Any product that may be evaluated in this article, or claim that may be made by its manufacturer, is not guaranteed or endorsed by the publisher.

## Supplementary Material

Supplementary Figure 1 Surgical atlas. **(A)** Continued T-shaped fashion and inverted T-shaped fashion in infundibulum and MPA incision to adequately release sub- and supra-valvular tissues. **(B)** Longitudinal incision along the middle line of two leaflets to the level of PVA is performed to release constraints on PVA in patients with bicuspid PV and ruler shows annulus extension. **(C)** A dilator is used to size the annulus to assure fully releasing and remained across PVA to avoid annular shrunk and restenosis by continuous sutures in AS and knotting. **(D)** Triangle and rectangle patches are used to enlarge main PA and RVOT.

Supplementary Figure 2 Pulmonary valve level short-axis section to show PV morphology. Panel **(A)** shows a case with abnormal PV morphology of bicuspid valve and Panel **(B)** demonstrates a normal morphology with tricuspid valve.

Supplementary Table 1 Univariable Cox proportional-hazards analyses for screening covariates introduced into a multivariable model.

Supplementary Table 2 Baseline characteristics of patients with TOF before and after propensity sore matching.

Supplementary Table 3 Clinical outcomes of patients with CR-TOF before and after propensity sore matching.

Supplementary Table 4 Risk of primary and secondary outcomes in the propensity-score-matched cohort.
