# Supplementary figures and images for: Association of Pulmonary Valve Morphology Differences With Outcomes in Tetralogy of Fallot Repair With Right Ventricular Outflow Tract Incision
Source: Front Cardiovasc Med. 2021 Aug 4;8:695876. doi: 10.3389/fcvm.2021.695876 (PMC8372408; doi:10.3389/fcvm.2021.695876)

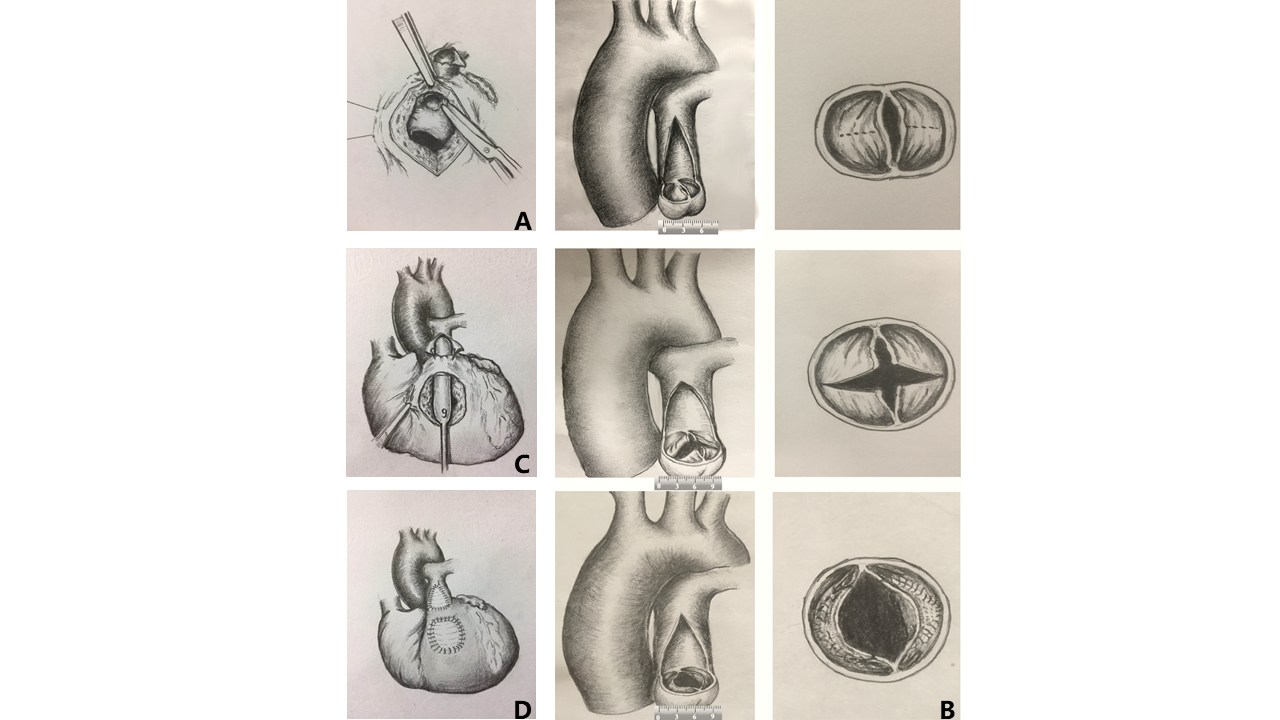

Supplement: Supplementary Figure 1 — Surgical atlas. (A) Continued T-shaped fashion and inverted T-shaped fashion in infundibulum and MPA incision to adequately release sub- and supra-valvular tissues. (B) Longitudinal incision along the middle line of two leaflets to the level of PVA is performed to release constraints on PVA in patients with bicuspid PV and ruler shows annulus extension. (C) A dilator is used to size the annulus to assure fully releasing and remained across PVA to avoid annular shrunk and restenosis by continuous sutures in AS and knotting. (D) Triangle and rectangle patches are used to enlarge main PA and RVOT. [file Image_1.TIF]

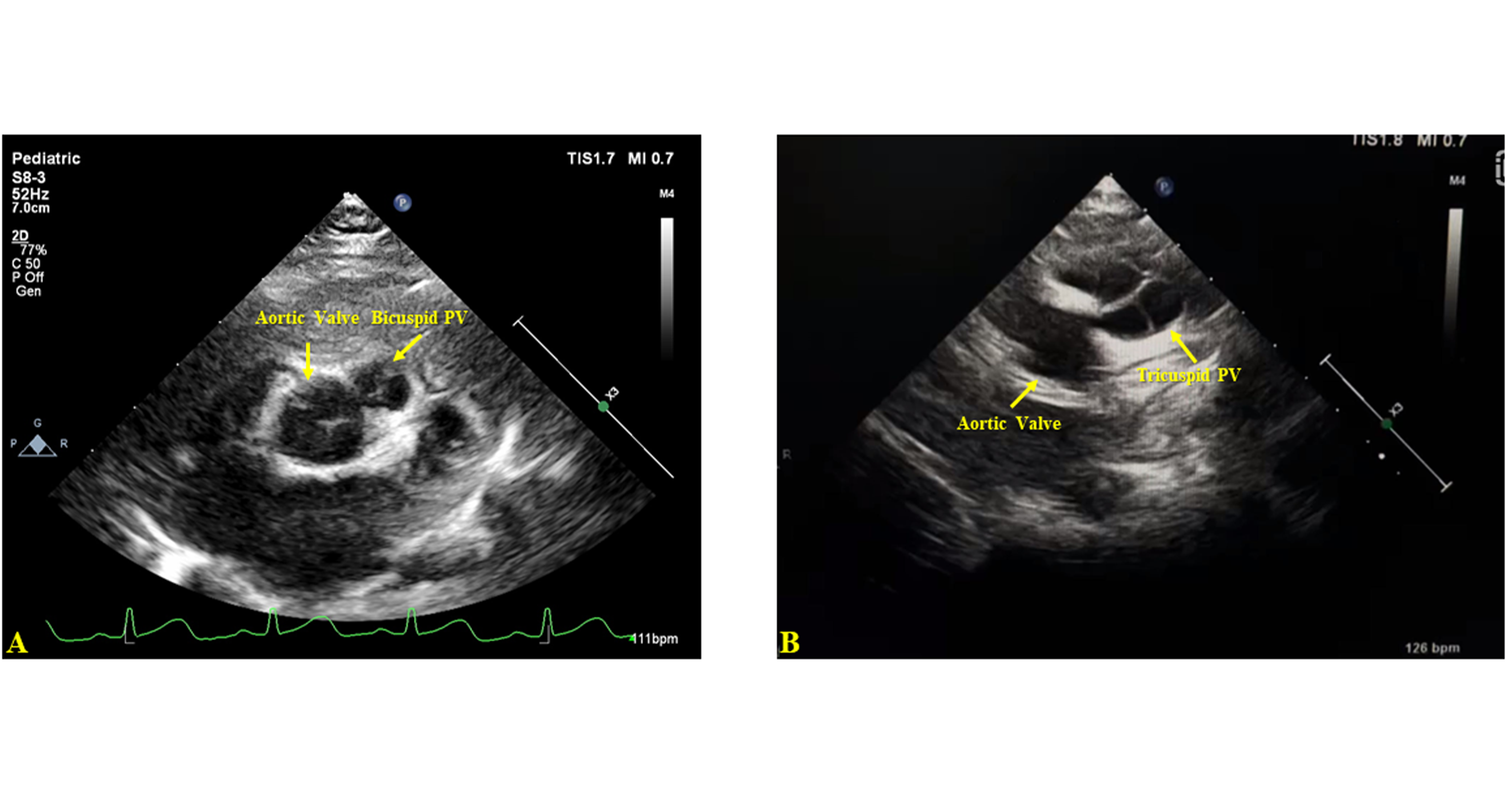

Supplement: Supplementary Figure 2 — Pulmonary valve level short-axis section to show PV morphology. Panel (A) shows a case with abnormal PV morphology of bicuspid valve and Panel (B) demonstrates a normal morphology with tricuspid valve. [file Image_2.TIF]
