# Supplementary material for: Association of Pulmonary Valve Morphology Differences With Outcomes in Tetralogy of Fallot Repair With Right Ventricular Outflow Tract Incision
Source: Front Cardiovasc Med. 2021 Aug 4;8:695876. doi: 10.3389/fcvm.2021.695876 (PMC8372408; doi:10.3389/fcvm.2021.695876)
Supplement: Supplementary Table 1 — Univariable Cox proportional-hazards analyses for screening covariates introduced into a multivariable model. [file Data_Sheet_1.docx]

**Table S1. Univariate Cox proportional-hazards analyses for screening covariates introduced into a multivariate model.**

|  | Statistics | *Primary outcome at follow up  HR (95%CI), P value | Significant APG at follow up  HR (95%CI), P value | Significant PR at follow up  HR (95%CI), P value | Reintervention  HR (95%CI), P value |
| --- | --- | --- | --- | --- | --- |
| Sex |  |  |  |  |  |
| Female, no. (%) | 671 (40.1) | 1.0 | 1.0 | 1.0 | 1.0 |
| Male, no. (%) | 1002 (59.9) | 0.96 (0.81-1.15)  P=0.67 | 0.99 (0.703-1.39)  P=0.95 | 0.99 (0.81-1.20)  P=0.92 | 0.61 (0.29-1.31)  P=0.20 |
| Age, days | 555.2 ± 747.6 | 1.00 (1.00-1.00)  P=0.11 | 1.00 (1.00- 1.00)  P=0.86 | 1.00 (1.00-1.00)  P=0.044 | 1.00 (1.00, 1.00)  P=0.05 |
| Height, cm | 75.6 ± 14.8 | 0.99 (0.98-1.00)  P=0.003 | 0.99 (0.98, 1.03)  P=0.14 | 0.99 (0.98, 1.00)  P=0.002 | 0.93 (0.88-0.97)  P=0.003 |
| Weight, kg | 9.9 ± 5.0 | 0.99 (0.97-1.01)  P=0.25 | 1.0 (0.97-1.03)  P=0.98 | 0.98 (0.96-1.0)  P=0.11 | 0.81 (0.67-0.99)  P=0.036 |
| Preop. Saturation, % | 83.7 ± 9.0 | 0.90 (0.98-1.00)  P=0.021 | 0.99 (0.97-1.01)  P=0.27 | 0.99 (0.98-1.00)  P=0.013 | 1.03 (0.98-1.08)  P=0.22 |
| PVA diameter, mm | 8.7 ± 2.5 | 0.85(0.82, 0.89)  P<0.001 | 0.86 (0.79, 0.93)  P<0.001 | 0.85 (0.81, 0.89)  <0.001 | 0.73 (0.60, 0.90)  P=0.002 |
| PVA Z-score | -2.1 ± 1.0 | 0.72 (0.65-0.79)  P<0.001 | 0.70 (0.58-0.85)  P<0.001 | 0.71 (0.64-0.79)  <0.001 | 0.68 (0.45-1.03)  P=0.07 |
| RVOT gradient, mmHg | 74.1 ± 16.8 | 1.01 (1.00- 1.01)  P=0.022 | 1.01 (1.00-1.02)  P=0.12 | 1.01 (1.00-1.01)  P=0.014 | 1.02 (0.99-1.04)  P=0.14 |
| Operator experience |  |  |  |  |  |
| Less experienced operator, no. (%) | 483 (28.8) | 1.0 | 1.0 | 1.0 | 1.0 |
| Experienced operator, no. (%) | 1190 (71.1) | 1.23 (1.02-1.49)  P=0.031 | 1.50 (1.01-2.2)  P=0.042 | 1.20 (0.97-1.48)  P=0.09 | 2.75 (0.95-7.97)  P=0.06 |
| PV morphology |  |  |  |  |  |
| Abnormal PV, no. (%) | 1488 (89.0) | 1.0 | 1.0 | 1.0 | 1.0 |
| Normal PV, no. (%) | 185 (11.0) | 0.45 (0.31-0.66)  P<0.001 | 0.17 (0.05-0.53)  P=0.002 | 0.54 (0.37-0.79)  P=0.001 | 0.00 (0.00-Inf)  P=1.00 |
| Surgical strategies |  |  |  |  |  |
| AS, no. (%) | 944 (56.4) | 1.0 | 1.0 | 1.0 | 1.0 |
| TAP, no. (%) | 729 (43.6) | 2.42 (2.02-2.91)  p<0.001 | 1.99 (1.41-2.81) 8  P<0.001 | 2.85 (2.31-3.51)  <0.001 | 4.35 (1.76-10.78)  P=0.002 |
| ASD/PFO |  |  |  |  |  |
| No, no. (%) | 1195 (71.4) | 1.0 | 1.0 | 1.0 | 1.0 |
| Yes, no. (%) | 478 (28.6) | 0.98 (0.81-1.19)  P=0.86 | 0.88 (0.60-1.28)  P=0.49 | 0.98 (0.80-1.21)  P=0.86 | 0.85 (0.36-2.00)  P=0.70 |
| PDA |  |  |  |  |  |
| No, no. (%) | 1497 (89.5) | 1.0 | 1.0 | 1.0 | 1.0 |
| Yes, no. (%) | 176 (10.5) | 1.49 (1.16-1.93)  P=0.002 | 2.09 (1.35-3.23)  p<0.001 | 1.27 (0.94-1.71)  P=0.12 | 2.70 (1.09-6.69)  P=0.032 |
| Staged operation |  |  |  |  |  |
| No, no. (%) | 1612 (96.4) | 1.0 | 1.0 | 1.0 | 1.0 |
| Yes, no. (%) | 61 (3.6) | 1.50 (0.97-2.30)  P=0.06 | 2.00 (0.98-4.09)  P=0.06 | 1.50 (0.93-2.41)  P=0.09 | 4.04 (1.21-13.44)  P=0.022 |

Data are presented as n (%) or mean ± standard deviation where appropriate. AS, annular-sparing; TAP, transannular patch; Pre-op, preoperative; PVA, pulmonary valve annulus; PVAD, pulmonary valve annulus diameter; ASD, atrial septal defect; PDA, patent ductus arteriosus; RVOT, right ventricular outflow tract; MPA, main pulmonary artery; LPA, left pulmonary artery; RPA, right pulmonary artery; MAPACs, major aortopulmonary collateral arteries.

*The operator experience is defined according to volume of complete repair of TOF they performed each year between 2012 and 2017.

**Table S2. Baseline Characteristics of Patients with TOF Before and After Propensity Sore Matching**

| Patients Characteristic | Before Matching | | |  | After Matching | | |
| --- | --- | --- | --- | --- | --- | --- | --- |
|  | Normal PV  (N=187) | Abnormal PV  (N=1501) | Standardized  Difference, % |  | Normal PV  (N=183) | Abnormal PV  (N=366) | Standardized  Difference, % |
| Male sex, no. (%) | 110 (58.8) | 901 (60.6) | 2.5 |  | 107 (58.5) | 229 (62.6) | 8.4 |
| Age, days | 311 (220-674) | 318 (224-524) | 16.8 |  | 311 (212-646) | 308 (213-497) | 1.7 |
| Height, cm | 78.4±18.2 | 75.2±14.3 | 19.4 |  | 76.8±14.7 | 75.3±16.4 | 9.5 |
| Weight, kg | 8.7(7.8-10.8) | 9.0 (7.6-10.5) | 12.0 |  | 8.6 (7.8-10.7) | 8.7 (7.5-10.0) | 0.4 |
| Preop. SaO_2_, % | 85.9±7.7 | 83.4±9.1 | 29.5 |  | 85.9±7.7 | 83.5±9.1 | 28.2 |
| PVAD, mm | 9.9±3.0 | 8.6±2.4 | 49.0 |  | 9.7±2.6 | 9.3±2.6 | 14.0 |
| PVA Z-score | -1.7 (-2.4-0.9) | -2.3 (-2.8-1.6) | 49.9 |  | -1.8 (-2.4-0.9) | -2.0 (-2.5-1.1) | 16.9 |
| Pre-op. RVOT peak gradient, mmHg | 71.1±18.9 | 74.4±16.5 | 18.7 |  | 71.2±18.9 | 73.5±16.8 | 12.9 |
| Procedures for complete repair |  |  | 73.5 |  |  |  | 3.7 |
| AS repair, no. (%) | 158 (84.5) | 787 (52.4) |  |  | 154 (84.2) | 303 (82.8) |  |
| TAP repair, no. (%) | 29 (15.5) | 714 (47.6) |  |  | 29 (15.8) | 63 (17.2) |  |
| Staged repair, no. (%) | 5 (2.7) | 56 (3.7) | 6.0 |  | 5 (2.7) | 7 (1.9) | 5.4 |
| Additional procedures |  |  |  |  |  |  |  |
| MPA plasty, no. (%) | 55 (29.4) | 872 (58.1) | 60.4 |  | 55 (30.1) | 145 (39.6) | 20.2 |
| LPA plasty, no. (%) | 11 (5.9) | 212 (14.1) | 27.7 |  | 11 (6.0) | 32 (8.7) | 10.5 |
| RPA plasty, no. (%) | 4 (2.1) | 48 (3.2) | 6.6 |  | 4 (2.2) | 10 (2.7) | 3.5 |
| PV plasty, no. (%) | 0 (0.0) | 24 (1.6) | 18.0 |  | 0 (0.0) | 4 (1.1) | 14.9 |
| MAPCAs closure, no. (%) | 4 (2.1) | 92 (6.1) | 20.1 |  | 4 (2.2) | 13 (3.6) | 8.2 |
| ^*^Operator experience |  |  | 1.5 |  |  |  | 0.6 |
| Experienced operator, no. (%) | 132 (70.6) | 1070 (71.3) |  |  | 130 (71.0) | 261 (71.3) |  |
| Less experienced operator, no. (%) | 55 (29.4) | 431 (28.7) |  |  | 53 (29.0) | 105 (28.7) |  |
| Follow-up duration, m | 49 (31-63) | 49 (36-64) | 11.8 |  | 49 (36-64) | 49 (30-63) | 9.2 |

Data are presented as n (%), mean ± standard deviation, or median (interquartile range [IQR]). AS, annular-sparing; TAP, transannular patch; Pre-op, preoperative; PVA, pulmonary valve annulus; PVAD, pulmonary valve annulus diameter; ASD, atrial septal defect; PDA, patent ductus arteriosus; RVOT, right ventricular outflow tract; MPA, main pulmonary artery; LPA, left pulmonary artery; RPA, right pulmonary artery; MAPACs, major aortopulmonary collateral arteries.

*The operator experience is defined according to volume of complete repair of TOF they performed each year between 2012 and 2017.

**Table S3. Clinical Outcomes of Patients with CR-TOF Before and After Propensity Sore Matching**

| Patients Characteristic | Before Matching | | |  | After Matching | | | |
| --- | --- | --- | --- | --- | --- | --- | --- | --- |
|  | Normal PV  (N=187) | Abnormal PV  (N=1501) | P-value |  | Normal PV  (N=183) | Abnormal PV  (N=366) | P-value |  |
| Crossclamp time, min | 73.4±26.2 | 75.9±26.7 | 0.24 |  | 72.9±26.1 | 73.0±24.9 | 1.0 |  |
| ^&^Repump, no. (%) | 1 (0.5) | 25 (1.7) | 0.24 |  | 1 (0.6) | 1 (0.3) | 1.0 |  |
| Ventilation duration, hours | 13 (8-23) | 18 (10-28) | <0.001 |  | 13 (8-23) | 15 (9-24) | 0.10 |  |
| ICU stay duration, days | 2 (1-4) | 3 (1-4) | 0.012 |  | 2 (1-4) | 2 (1-4) | 0.62 |  |
| Postop length of stays, days | 8 (7-12) | 9 (7-12) | 0.15 |  | 8 (7-12) | 8 (7-11) | 0.93 |  |
| In-hospital death, no. (%) | 2 (1.1) | 13 (0.9) | 0.78 |  | 2 (1.1) | 0 (0.0) | 0.11 |  |
| APG in TPS Class 3 at discharge, no. (%) | 8 (4.3) | 96 (6.4) | 0.25 |  | 8 (4.4) | 27 (7.4) | 0.17 |  |
| PR in TPS Class 3 at discharge, no. (%) | 11 (5.9) | 174 (11.6) | 0.018 |  | 11 (6.0) | 28 (7.7) | 0.48 |  |
| Reintervention at follow up, no. (%) | 0 (0.0) | 27 (1.8) | 0.07 |  | 0 (0.0) | 4 (1.1) | 0.31 |  |
| *Adequacy of PV repair at follow up |  |  |  |  |  |  |  |  |
| TPS for APG |  |  | 0.002 |  |  |  | 0.07 |  |
| Class 1 (APG＜20 mmHg) , no. (%) | 116 (62.7) | 841 (56.5) |  |  | 113 (62.4) | 230 (62.8) |  |  |
| Class 2 (APG 20-40 mmHg), no. (%) | 66 (35.7) | 512 (34.4) |  |  | 65 (35.9) | 115 (31.4) |  |  |
| Class 3 (APG＞40 mmHg) , no. (%) | 3 (1.6) | 135 (9.1) |  |  | 3 (1.7) | 21 (5.8) |  |  |
| TPS for PR |  |  | <0.001 |  |  |  | 0.004 |  |
| Class 1 (none/trivial PR), no. (%) | 89 (48.1) | 449 (30.2) |  |  | 86 (47.5) | 121 (33.1) |  |  |
| Class 2 (mild/mild-moderate PR), no. (%) | 68 (36.8) | 644 (43.3) |  |  | 67 (37.0) | 178 (48.6) |  |  |
| Class 3 (moderate or greater PR), no. (%) | 28 (15.1) | 395 (26.6) |  |  | 28 (15.5) | 67 (18.3) |  |  |
| TR Moderate or greater at follow up, no. (%) | 2 (1.08) | 17 (1.14) | 0.94 |  | 2 (1.1) | 1 (0.3) | 0.26 |  |
| ^#^ Primary outcome at follow up | 29 (15.7) | 486 (32.7) | <0.001 |  | 29 (16.0) | 85 (23.2) | 0.05 |  |

Data are presented as n (%), mean ± standard deviation, or median (interquartile range [IQR]).

ECMO, corporeal membrane oxygenation; ICU, intensive care unit; postop, postoperative; TPS, technical performance score; APG, peak annular gradient; PR, pulmonary regurgitation; Other abbreviations as above.

^&^Repump means patients required a return to bypass during index operation.

^*^Patient numbers in adequacy of PV repair at follow up excluded in-hospital deaths.

^#^The primary outcome was a composite of reintervention or TPS in Class 3 (APG＞40 mmHg or moderate or greater PR).

**Table S4 Risk of Primary and Secondary Outcomes in the Propensity-Score-Matched Cohort.**

| Analysis | Primary outcome* | Significant APG |
| --- | --- | --- |
| No. of events/ no. of patients at risk (%) |  |  |
| Normal PV | 29/185 (15.7) | 3/185 (1.6) |
| Abnormal PV | 486/1488 (32.7) | 135/1488 (9.1) |
| Propensity Score Matching analysis^&^ — hazard ratio (95% CI) | 0.80 (0.52, 1.24) | 0.28 (0.08, 0.97) |

Events (%) and HRs from adjusted analyses are presented. HRs were calculated in reference to the abnormal PV group.

CI, confidence interval; HR, hazard ratio; Other abbreviations as above.

*The primary outcome was a composite of reintervention or TPS=Class 3 (APG＞40 mmHg, moderate or greater PR, or both).

^&^Shown is the hazard ratio from a multivariable Cox model (adjusted for age, height, preoperative saturation, PVA diameter, PVA z-score, RVOT gradient, operator experience, surgical strategies and staged operation) with the same strata and covariates with matching according to the propensity score. The analysis included 549 patients (183 who had normal PV and 366 who did not).
